# Sleep, circadian, and mental health in neurodivergent neurotypes: lived experience perspective on the research landscape and roadmaps

**DOI:** 10.1093/sleep/zsag183

**Published:** 2026-07-04

**Authors:** Grace Gatera, Jess Lanham, Sarah Laxhmi Chellappa

**Affiliations:** Young Leaders for Global Mental Health, Kigali, Rwanda; School of Psychology, Faculty of Environmental and Life Sciences, University of Southampton, Southampton, Hampshire, United Kingdom; School of Psychology, Faculty of Environmental and Life Sciences, University of Southampton, Southampton, Hampshire, United Kingdom

**Keywords:** sleep, insomnia, circadian rhythms, delayed sleep–wake phase syndrome, cognitive behavioral therapy for insomnia, mental health, quality of life, autism, ADHD, neurodivergent

## Abstract

Neurodivergent neurotypes refer to brains that function differently from what is considered the societal standard or “stereotypical” norm, and include autism and attention deficit hyperactivity disorder (ADHD). Currently, global estimates suggest that 15 per cent to 20 per cent of the global population is neurodivergent. Autistic people and those with ADHD often experience mental health problems and a heightened likelihood of anxiety and depression. Epidemiological evidence shows that sleep and circadian rhythm disruption, such as low self-reported sleep quality, insomnia symptoms, and delayed sleep–wake phase disorder, are common in autistic people and those with ADHD. Despite scientific advancements, a comprehensive framework integrating sleep and circadian factors associated with mental health problems in neurodivergent neurotypes remains undeveloped. This perspective, written by people with lived experience of neurodivergent neurotypes, examines mental health problems, particularly anxiety, depression, and suicide risk, in autistic people and those with ADHD; sleep and circadian rhythm disruption in these neurotypes; and the growing epidemiological evidence linking sleep, circadian, and mental health problems in these neurotypes compared to people with neurotypical development. Our perspective advocates for an integrated biopsychosocial model that accounts for the interplay between sleep, circadian rhythms, and mental health in neurodivergent neurotypes. Moreover, it identifies gaps and future research avenues, and the need for personalized behavioral interventions to improve their sleep, circadian, and mental health problems. Co-production of research is advocated as a method to ensure that people with neurodivergent neurotypes are included as equal partners in the entire research process, as a means to best support their sleep, circadian, and mental health.

## Perspective

### Paradigm shift in neurodivergent research priorities

Recent evidence focusing on neurodivergent (i.e. attention deficit hyperactivity disorder [ADHD], autism) research priorities indicates a mismatch between what the research focuses on and what neurodivergent people want to see researched [1–3]. Whereas increased funding and publications are shifting towards research on mental health services and lifespan issues, there is still a dominant focus on biological research (e.g. genetic, animal experimental models) and pharmacological intervention studies [1–3]. While the latter is essential for understanding factors underlying, e.g. sleep and circadian problems observed in autistic mouse models, direct translation for mental health conditions and quality of life in autistic people and those with ADHD is limited. This reinforces the need for an increase in participatory research whereby researchers work collaboratively with autistic people and those with ADHD to foster a neurodiversity-focused paradigm. Such co-participatory research indicates that, among the top five research priorities, are mental health, quality of life, and support/educational services [1–3]. Similarly, a recent co-participatory study focusing on potential research priorities in the area of mental health, sleep and circadian rhythms indicated that a top-ten priority should be “What is the relationship between the body clock and mental health in neurodivergent individuals and does body clock disruption worsen mental health in these individuals?” [2]. This question in particular highlights the need to better understand circadian mechanisms underlying sleep and mental health problems in neurodivergent people, which is a research priority that has been largely overlooked. Collectively, these questions resonate with the lived experts involved in this perspective who share similar experiences that point towards such research priorities for future human studies (Table 1). In this perspective, we discuss what is currently known about sleep/circadian and mental health problems in neurodivergent neurotypes, what are the current research gaps, and what future studies could be conducted within a neurodiversity-focused paradigm to directly support quality of life in neurodivergent people.

**Table 1:** Lived experiences of sleep and mental health concerns, and research priorities in neurodivergent people

1. “As a neurodivergent student, school was extremely challenging, and it had dire effects on my mental and physical health. I was experiencing high levels of anxiety due to sensory sensitivities, and my sleep was greatly affected. Heightened anxiety often left me ruminating late at night or struggling to fall asleep. I was unable to catch up on the missed sleep because I had to wake up early for school the next day.” 2. “My anxiety reduced my sleep, but the reduction in sleep also increased my anxiety. My sleep and mental health problems have worsened with age. Due to a late diagnosis, the support I received was minimal. In turn, my quality of life was affected, leading to depression and reduced physical and mental health, with an inability to function on a day-to-day basis.” 3. “I want to see co-participatory research with lived experts that explores how improved support systems (e.g. mandatory training) affect the sleep/biological cycles and mental health of neurodivergent individuals, using focus groups and self-report measures to enhance their educational experiences and quality of life.” 4. “I have lived with anxiety and depression since childhood, and I have recently begun to understand myself as neurodivergent. Many everyday things that other people seem to manage easily have been difficult for me, like situations that may seem simple socially, leaving me needing a lot of time to recover, or understanding what my body is telling me and coping with busy or overwhelming sensory feelings. I have had long-term trouble sleeping since I was young. This can lead me to either push myself too hard or not respond enough to what my body needs.” 5. “These experiences show why research in this area is so important. The main questions for me are practical: what actually helps people get through everyday life, feel less overwhelmed, and live with dignity? I want to understand what kind of support, environmental changes, and ways of delivering services make life easier, without expecting people to hide who they are or act differently to fit in.” 6. “I want to see research that includes people with lived experience from the very beginning. Helpful approaches could include tracking people’s experiences over time, conducting interviews to understand what really matters to people, and exploring real-life settings, such as schools, workplaces, and homes. It is also important that research include people from many different backgrounds, ages, cultures, and communication styles, so that the findings reflect the real diversity of neurodivergent experiences.” 7. “I cannot tolerate artificial light as I get headache, and I can hear the sound they make. I avoid rooms with bright lights, and get little light during the day. I also spend a lot of time on my phone socializing, as it feels safer than meeting up with people. I catch myself at 02:00 am. using my phone on social media. I end up sleeping very late because of it, and feel it is affecting my mental health. I want to see co-participatory research on why light affects sleep and mental health, and what can be done to help the quality of life of neurodivergent people.” 8. “I often forget to eat when I study and work, and end up eating a lot all at once at the end of the day. This gets even worse when I experience anxiety and depression. Next thing I know, I cannot sleep well because I feel too full, end up with insomnia, and feel groggy and tired when I wake-up. Many neurodivergent people experience this, but it seems to be less common among neurotypical people. As a “fix”, I set a timer to remind me to eat, but find this stressful as well. I do not know why this happens to me, and wonder why eating and sleep affect me so much, and what strategies can meaningfully help neurodivergent people.”

### A “mental health crisis” among neurodivergent neurotypes

Autistic individuals (identity-first in accordance with language preference within the autistic community [4]) and those with ADHD often experience a higher risk of mental health problems, particularly anxiety, depression, and suicide ideation/attempts, compared to individuals with neurotypical development [5]. Surveys from people with lived experience of these neurodivergent neurotypes indicate that ~80 per cent have mental health problems (e.g. anxiety, depression, and low psychological wellbeing) during their lifespan, while only 20 per cent report being able to access counseling services [5]. Meta-analysis of 96 studies (~5000 autistic and ~10000 individuals with neurotypical development) has shown an overall pooled prevalence estimate of 20 per cent for anxiety disorders, 12 per cent for impulse-control/conduct disorders and depressive disorders, which are ~3-fold higher than those for the general population [6]. Similarly, a meta-analysis of 18 cross-sectional studies (1615 individuals with ADHD and 3128 individuals with neurotypical development) showed a fourfold increase in the association between ADHD and depression among young adults (18–30 years) [7]. Importantly, recent evidence indicates a pooled prevalence of ~25 per cent for suicide ideation, and ~9 per cent for suicide attempts in young autistic people (10–25 years) [8], with similar estimates for young people (10–19 years) with ADHD [9]. Notwithstanding the high prevalence of mental health problems in autistic people and those with ADHD, and their impact on public health and quality of life [10], daily biological factors contributing to these associations remain to be fully established, including the contribution of sleep and circadian rhythms.

### Sleep and circadian challenges among neurodivergent neurotypes

Sleep and circadian rhythm disruption frequently occur in autistic people across the lifespan, as compared to age-matched people with neurotypical development [11]. Meta-analysis including eight studies (194 autistic adults and 277 adults with neurotypical development) indicates that autistic adults had ~20 per cent lower self-reported sleep quality and sleep efficiency, compared to age- and sex-matched people with neurotypical development [12]. Moreover, field-based and laboratory studies in adolescents and young adults (13-25 years) indicate objectively-assessed sleep problems (i.e. wrist-worn wearables and/or polysomnography recordings), including longer sleep onset latency and wake after sleep onset. Additionally, ~30 per cent of young autistic people (10–25 years) experience insomnia complaints, relative to age- and sex-matched young people with neurotypical development [13]. Up to a third of autistic children and adolescents [14] as well as autistic adults [15] report later circadian preference, which is up to two-fold higher compared to age-matched individuals with neurotypical development. In a similar vein, delayed sleep–wake phase is prevalent in young autistic adults (18–35 years old), with a pooled estimate of 36 per cent [16]. Of note, a case–control study using ambulatory circadian monitoring indicates a delayed sleep midpoint in autistic adults based on wrist-worn indexed activity, which may suggest a delayed circadian phase within this age group [17]. Such circadian disturbances could be due to biological factors, such as melatonin processing differences, potentially resulting in attenuated melatonin secretion and/or delayed dim light melatonin onset phase, and genetic vulnerability to sleep and circadian rhythm disruption, but also psychosocial and environmental factors, such as increased evening/nighttime screen time (Figure 1) [18,19]. Sleep and circadian rhythm disruption are similarly common in people with ADHD, with 25%–40% of adults with ADHD self-reporting sleep disorders, particularly insomnia and sleep-related breathing disorders [20]. A meta-analysis from 16 cross-sectional studies (722 children with ADHD and 638 age- and sex-matched children with neurotypical development) shows more sleep onset difficulties, nighttime awakenings, and daytime sleepiness (small to medium effect sizes) in children with ADHD [21]. Similarly, a meta-analysis of 13 cross-sectional studies revealed that adults with ADHD had longer sleep onset latency and lower sleep efficiency (medium effect size), relative to age-matched people with neurotypical development [22]. Moreover, there is an established association between ADHD traits and circadian disturbances, with up to ~30 per cent of adults with ADHD reporting late circadian preference, delayed circadian phase (as indexed by dim light melatonin onset), and higher likelihood of delayed sleep–wake phase syndrome (medium effect size) [23]. These findings align with the experiences described by people with lived experience of neurodivergent neurotypes who appear to have more “body clock” dysregulation compared to individuals with neurotypical development (Table 1). Moreover, a recent priority setting partnership of individuals with lived experience, carers, clinicians, and researchers indicates that understanding the relationship between circadian rhythms and mental health in neurodivergent people, and how circadian disruption may worsen mental health are among the top-ten priorities in mental health research [2].

**Figure 1:**
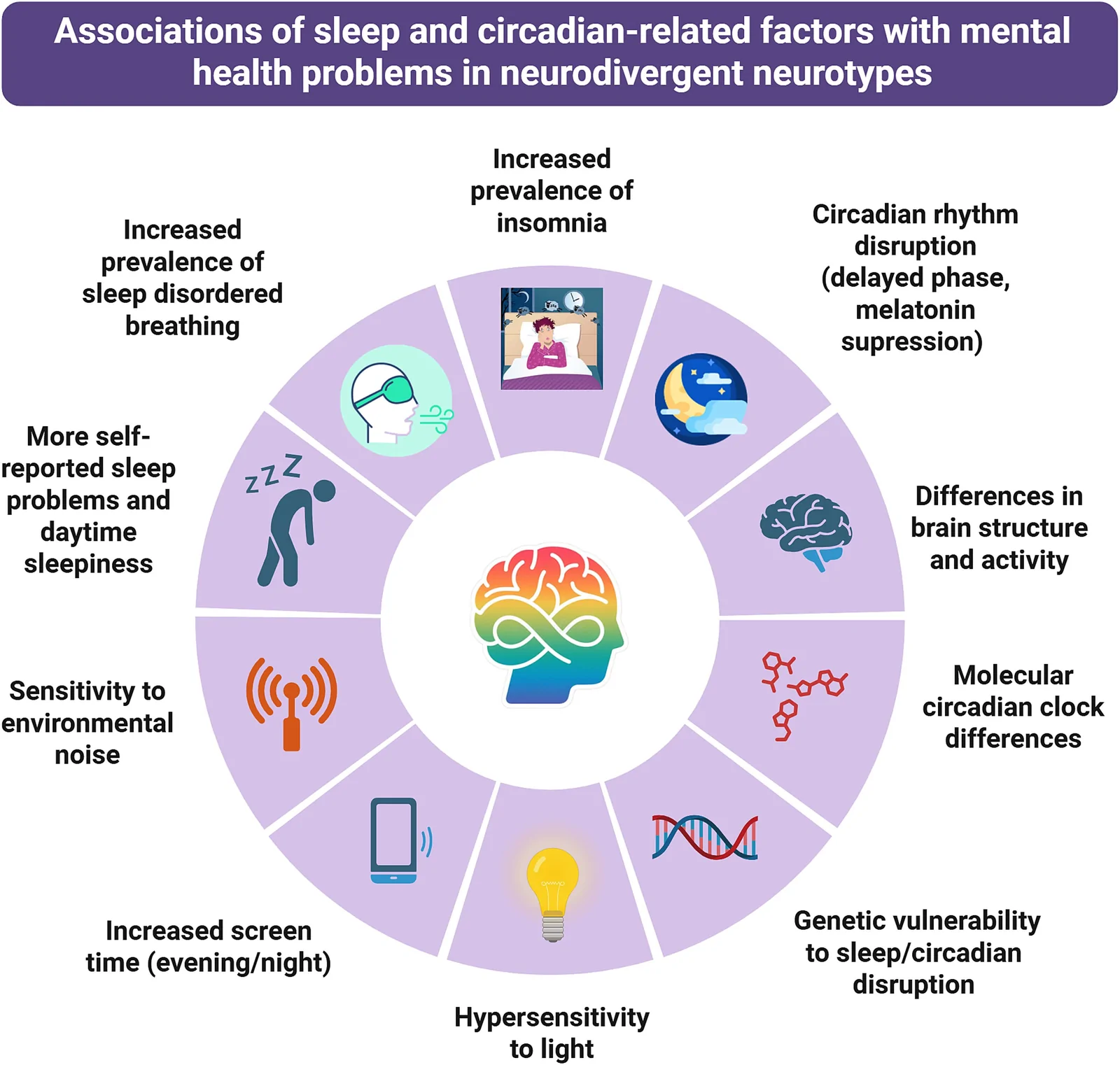
Framework of the associations between sleep-circadian related risk factors and mental health conditions (e.g. anxiety, depression) in neurodivergent neurotypes (e.g. autism, ADHD).

### Is the “mental health crisis” among neurodivergent neurotypes linked to sleep and circadian problems?

Disturbances in sleep and circadian rhythms are bidirectionally intertwined with almost every category of mental disorder [24, 25]. Sleep and circadian rhythm disruption predict the onset of mental disorder, and are one of the earliest signs of relapse [26]; they occur during and between episodes of mental illness, and the intensity of sleep and psychiatric disturbances typically co-vary [27]. Moreover, pharmacological and psychological treatments of sleep disturbances can alleviate psychopathology, and vice-versa. These transdiagnostic associations between sleep and circadian rhythm disruption and mental health problems may underscore a heightened vulnerability in autistic people and those with ADHD.

Emotional (i.e. anxiety, depression, social withdrawal) and behavioral differences (i.e. somatic complaints, rule-breaking, aggression) in young autistic people (8–16 years) with self-reported sleep problems (e.g. bedtime resistance, daytime sleepiness) are associated with more autistic traits [28]. Similarly, recent evidence indicates that autistic traits in middle-aged and older adults were associated with higher depression symptom severity, lower quality of life, insomnia severity, and lower self-reported sleep quality [29]. Additionally, insomnia severity and late chronotype partly mediated the association of autistic traits with depression symptom severity and with quality of life [29]. Network analysis exploring the interactional pattern between autistic traits, sleep problems, and emotional and behavioral differences in autistic children (5–13 years) with self-reported sleep problems shows an association of highly connected network, with depression, anxiety, and behavioral differences as most influential in the network [30]. This could signify shared underlying pathways/commonalities contributing to sleep and mental health conditions in this population, based on their autistic trait differences. Not surprisingly, similar associations of sleep and circadian rhythm disruption and mental health problems (e.g. anxiety and depression) have been observed in people with ADHD. Compared to those who did not report sleep problems, adolescents with ADHD (13–17 years) and self-reported sleep problems had a 2-3-fold higher likelihood of co-occurring depression and generalized anxiety disorder [31]. In middle-aged and older adults (50–60 years), ADHD traits were associated with higher depression symptom severity, lower quality of life, insomnia severity, lower self-reported sleep quality, and a later circadian preference [32]. Importantly, insomnia symptom severity mediated the association of higher ADHD traits with lower quality of life.

### Gaps of knowledge and roadmaps: The need for new co-participatory basic and clinical research for neurodivergent neurotypes

In a stark contrast to sleep, circadian and mental health research conducted in neurotypical people, there has been a historic dearth of research in neurodivergent individuals, largely due to assumptions that the findings in neurotypical populations (“general population”) are directly translatable to neurodivergent people. Thus, there is currently no unified biopsychosocial frameworkto explain the higher likelihood of sleep, circadian, and mental health disruptions in neurodivergent neurotypes. Our knowledge is currently based on strands of separate epidemiological evidence, whereby (1) self-reported and objectively-assessed sleep and circadian disruption are known to be increased in autistic individuals and those with ADHD, (2) sleep and circadian disruption are bidirectionally associated with depression and anxiety, and (3) autistic individuals and those with ADHD often experience more depression, anxiety, and lower quality of life. However, there are no mechanistic insights from human studies that directly link these three strands of epidemiological evidence. This gap of knowledge needs to be addressed in future human studies to help understand this higher risk. Given that sleep and circadian factors evolve with developmental age and are shaped by biological sex [33], lifestyle, environmental influences, and brain maturation, future research should integrate these variables to inform more personalized and effective therapeutic support for autistic children and those with ADHD.

### Neurodivergent lived experience daily concerns

One outstanding question with direct lived experience relevance is the impact of environmental light exposure on sleep and circadian rhythms. Many neurodivergent people experience photophobia (i.e. sensory aversion to and overload from bright lights), with up to 70 per cent of individuals with ADHD self-report light hypersensitivity (i.e. discomfort or pain from light sources) [34], leading them to avoid sunlight and stay indoors during the day (where they experience dimmer light). Moreover, differences in executive function and temporal perception in autism and ADHD can cause individuals to lose track of time and/or forget to initiate their wind-down routines, increasing exposure to nighttime light. The combined net effect of this behavior can result inappropriately timed light exposure (e.g. increased exposure to evening/nighttime screen use, light avoidance during daytime). Light is the primary input to the circadian “clock”, such that daytime light phase advances circadian rhythms, and improves sleep quality and mood levels [35–37], whereas nighttime light disrupts them [37–40]. Because neurodivergent lived experience highlights that sensory sensitivities and time blindness may alter light exposure patterns compared to neurotypical people, it is possible that such differences in light exposure may explain the previously described associations of disrupted mood, sleep, and circadian rhythms. Another reported lived experience concern is that neurodivergent neurotypes often experience differences in sensory processing and perception (e.g. light, noise) compared to individuals with neurotypical development. However, it remains currently unknown whether such daily environmental risk factors affect the link between sleep and circadian rhythm disruption and mental health problems in this population.

Additionally, neurodivergent people frequently report fluctuating sleep-circadian and mental health needs as they age (Table 1), possibly due to the complex interplay of circadian rhythms, melatonin production, and sensory processing capabilities that are inherently dynamic, shifting due to natural aging processes, hormonal changes, and evolving life transitions. For instance, neurodivergent adults often experience “burnout,” where the long-term energy required to navigate a neurotypical world takes a physical and mental toll. This can lead to increased sensory sensitivities and sleep disturbances that differ drastically from childhood experiences [19]. Thus, there is a need to integrate a developmental lens as it can help clarify how sleep–circadian–mental health relationships differ across developmental age stages. Currently, there is limited human longitudinal and/or experimental evidence that allows insights into this developmental understanding, as 1. The majority of studies have historically focused on autistic and ADHD pediatric populations, and 2. Most studies are cross-sectional comparisons across developmental stages that provide partial insight. Future studies are needed to better understand how developmental age affects the underlying mechanisms for sleep, circadian, and mental health disruption in neurodivergent neurotypes, as well as to establish personalized behavioral interventions aimed at improving their sleep, circadian, and mental health.

### Potential behavioral interventions to improve mental health

#### Sleep-related interventions

Currently, one of the very few non-pharmacological approaches targeting sleep health in autistic adults is cognitive behavioral therapy for insomnia (CBT-I). Pilot findings on digital CBT-I suggest it may improve insomnia symptoms even after 6 months of post-randomization in autistic adolescents (up to 18 years) [41]. Currently, CBT-I has not been formally tested in those with ADHD. However, there is recent evidence that a transdiagnostic sleep and circadian intervention, combining cognitive behavioral therapy for insomnia with chronotherapy, can have beneficial effects in people with ADHD [42]. For instance, it improved sleep quality, reduced insomnia severity, and increased well-being, personal recovery, work ability, and perceived overall health from baseline to week 6 in people with sleep problems and depression, ADHD, and/or bipolar disorder compared to the control group [42]. Recently, digital CBT-I (in individuals with neurotypical development) has been shown to improve insomnia and depressive symptoms 10 weeks post-randomization, compared to a control sleep hygiene education, albeit without affecting face emotional processing [43]. These findings suggest an avenue for future research by applying digital CBT-I to improve both sleep and mental health problems in neurodivergent neurotypes. Of note, evidence-based strategies—including behavioral therapies, sleep habit practices, and, when appropriate, medication- are currently the only tested and effective approaches for improving sleep and circadian regulation in neurodivergent neurotypes [19]. Such interventions are generally low-risk, adaptable to individual needs, and have been shown (in some pilot studies) to help alleviate sleep disturbances while supporting cognitive and emotional well-being in these individuals. Future studies with randomized clinical trials using, e.g. digital CBT-I, are needed to establish their efficacy in the treatment of sleep and mental health with adaptations to the needs, preferences, and lived experiences of autistic people and those with ADHD. For instance, simplifying language, shortening sessions, and using digital CBT-I (avoiding face-to-face interaction and eye contact), and increasing material repetition will likely foster interest in these approaches and reduce potential attrition rates [43]. Lastly, other sleep-related interventions (e.g. acoustic stimulation of slow-wave activity, non-invasive neurostimulation targeting slow-wave sleep and EEG activity [26]) are yet to be formally tested in autistic individuals and those with ADHD.

#### Circadian-related interventions

Despite growing interest, a striking gap remains in research exploring the mechanisms by which circadian biology differentially affects neurodivergent neurotypes, which could help understand why up to a third of them experience later circadian preference and/or delayed sleep–wake phase syndrome. Indeed, the reported sensory sensitivity and avoidance to light exposure is likely to have a bi-directional relationship with circadian rhythms of melatonin, which reinforces the need to better understand what biological pathways (e.g. within the retina, downstream mood-related cortical regions) are affected in neurodivergent neurotypes. However, no human studies that have yet investigated whether autistic people and those with ADHD, or both, respond differently to light exposure compared with neurotypically developing peers, which limits the ability to establish light sensitivity as a modifiable factor implicated in sleep and circadian rhythm disruption in these neurodivergent neurotypes. This research question circles back to the Top 10 sleep-circadian questions related to basic research (rather than clinical intervention), reflecting a primary desire for people with lived experience for an improved understanding of circadian and mental health dysfunction among stakeholders. To address this gap, comprehensive, multimodal approaches that assess sleep and circadian rhythms concurrently are needed to advance mechanistic understanding and inform the development of personalized sleep/circadian behavioral support systems to promote their mental health.

### Lived experience paradigm shift toward participatory sleep-circadian intervention research

Neurodivergent people, including those with ADHD, autism, and other identities, disproportionately experience circadian rhythm differences and sleep disruption, but not always in ways “conventional” research captures. Lived experience will contribute to research what many neurodivergent people already know, but which is not adequately covered. Irregular sleep is not simply a “bad habit” or an “isolated symptom”. Among other things, it could be tied to sensory processing, interoceptive awareness, hyperfocus patterns, co-occurring anxiety, or even the chronic exhaustion of social masking. Moreover, lived experience and participatory research help to reframe what to target: not just sleep onset or duration, but the nervous system conditions that make sleep feel unsafe or inaccessible. Success cannot be defined as conforming to a neurotypical sleep schedule; it must be defined as feeling rested and restored enough to function and participate in life on neurodivergent levels, not on neurotypical ones. *This is what lived experience brings to the table.*

Specifically, there is a dearth of research that explores the efficacy of sleep-circadian interventions in neurodivergent populations, as most research was and still is formulated from neurotypical samples. Shifting towards participatory research to develop behavioral interventions can provide insight into the mechanisms underlying sleep-circadian problems in neurodivergent individuals. This will eventually lead to the formulation of sleep-circadian interventions that target these mechanisms and effectively support sleep and circadian problems in neurodivergent populations. Importantly, neurodivergent research on sleep interventions needs to move beyond the core components of CBT-I and explore other areas. When receiving support for sleep issues, there are currently no recommendations on, e.g. the impact of meal timing and/or physical activity. Both the timing and type of food can affect sleep, circadian rhythms and mental health [26]. Neurodivergent people often report that they eat late due to challenges in interoception (i.e. missed hunger cues in autistic people and those with ADHD), executive dysfunction making meal preparation a daunting task (particularly in those individuals who also have dyspraxia [developmental motor coordination disorder]), and time perception (e.g. forgetting what time to exercise in people with ADHD) [44]. These concerns frequently result in “forgetting” to eat throughout the day and frequently fuelling up in the evening/nighttime. Because current sleep recommendation for neurodivergent people do not factor meal timing as core components, these neurodivergent concerns remain unaddressed. Thus, participatory research would provide insight that are/are not supportive for neurodivergent individuals, allowing future interventions to be adapted or developed to better suit the individual needs of neurodivergent people.

Critically, participatory research in sleep and circadian behavioral interventions will finally halt the assumption of a “neurotypical baseline”, by bringing to light what some standard protocols misunderstand. For example, rigid bedtime routines that clash with demand avoidance, light therapy formats that trigger sensory overload, lack of recommendations about appropriate food content and timing, and/or psychoeducation delivered in ways that do not match diverse communication (e.g. non-verbal, verbal) and information processing styles. Participatory co-design will provide solutions to interventions to be flexible and modular: offering multiple entry points, sensory-adaptable formats, and pacing that accommodates executive function differences. It will also aid researchers in recognizing that sustainable sleep-circadian health for neurodivergent people will require systemic changes like school and workplace flexibility, not just individual behavioral "fixes".

## Conclusions

From a lived-experience perspective, we believe it is important that future studies move beyond a narrow clinical focus and reflect the realities of daily life. This includes paying closer attention to how sleep and circadian challenges shape support systems available in educational settings, work, relationships, and stress in practical terms. Longitudinal studies across development, rather than concentrating exclusively on childhood, are needed, such that we can better understand how these experiences shift across the lifespan. It feels especially important to continue to emphasize inclusion, by conducting human studies with people with intersectional identities, including people who do not use verbal speech, those with intellectual disabilities, and individuals from different cultural and socioeconomic contexts [10]. Importantly, lived experience should not be narrowly defined: People with lived experience should be engaged as co-researchers and co-producers, with openness around how they contribute, rather than being confined to specific roles or stages of the research process. Advocating for a more open, multi-sectoral, and multidisciplinary approach would allow lived experience to be included in ways that feel meaningful and self-defined. While these shifts require ongoing and, at times, difficult conversations, particularly around language, roles, expectations, and power, they can help reframe how neurodivergent research is constructed from the very roots and galvanize a new way to do research that directly impacts the quality of life of neurodivergent people.
